# School-Based Interventions to Prevent Overweight in Latin America: A Scoping Review and Policy Analysis

**DOI:** 10.3390/nu17213435

**Published:** 2025-10-31

**Authors:** Analí Morales-Juárez, Norma Alfaro, Yvette Fautsch-Macías, Maaike Arts, Paula Veliz, María F. Kroker-Lobos

**Affiliations:** 1 Research Center of the Prevention of Chronic Diseases, Institute of Nutrition of Central America and Panama, Guatemala City 1188, Guatemala; analimoralesj5@gmail.com; 2 Technical Unit for Human Resources Development, Institute of Nutrition of Central America and Panama, Guatemala City 1188, Guatemala; nalfaro@incap.int; 3 United Nations International Children’s Emergency Fund UNICEF Latin America and Caribbean Regional Office, Panama P.O. Box 0843-03045, Panama; yfautsch@unicef.org (Y.F.-M.); marts@unicef.org (M.A.); pveliz@unicef.org (P.V.)

**Keywords:** school-based interventions, overweight intervention outcomes, policy making, review

## Abstract

Overweight, including its severe form obesity, among children and adolescents has risen rapidly in Latin America. Schools play a critical role in addressing this growing public health challenge, as they offer a structured setting to implement preventive interventions targeting nutrition literacy, physical activity, and the food environment. The aim of this article is to describe the effectiveness of school-based interventions for preventing overweight in Latin America and whether existing policies, programs and other initiatives in the region align with the best available evidence. Among the 27 interventions included, most were conducted in Chile (41%), used a pre–post design (41%), adopted a preventive approach (85%), and reported positive effects (52%). Effective interventions included activities on nutrition literacy, physical activity, nutritious foods and diets, provision of free and safe drinking water (e.g., water that is free from microbial contamination and suitable for drinking), and healthy food environment. Experimental studies showed that the duration of effective interventions ranged from two months to two years and were primarily directed at primary school students including parents and teachers. Argentina, Chile, Colombia, Mexico, Peru, and Uruguay had multi-component policies and programs under a regulatory framework (e.g., laws or regulations passed by a government) based on the best available evidence to prevent overweight in school-aged children and adolescents. Only a limited number of countries have implemented these interventions. Ensuring program sustainability is critical to inform evidence-based childhood overweight prevention policies in the region. Policymakers should use the best scientific evidence to guide childhood overweight prevention strategies.

## 1. Introduction

In recent decades, the prevalence of overweight and its severe form, obesity: defined for children and adolescents aged 5–19 years by Body Mass Index (BMI)-for-age z-scores from the World Health Organization (WHO) Growth Reference, with overweight classified as >+1 standard deviation (SD) and obesity as >+2 SD above the WHO Growth Reference median [1]; has increased considerably across all age groups, including children and adolescents [2]. A study examining trends from 1990 to 2021 reported that among children and adolescents aged 5–14 years in Latin America (LatAm), the prevalence of overweight increased by 59%, while the prevalence of obesity rose by 265% [3]. Between 1990 and 2022, the age-standardized prevalence of obesity among school-aged children and adolescents (5–19 years) exceeded 20% among girls in 21 countries (11%) and boys in 35 countries (18%), with several of these countries located in LatAm [2].

Childhood overweight leads to profound health, social, and economic consequences. The health impacts include an increased risk of developing type 2 diabetes, hypertension, dyslipidemia, and cardiometabolic disease during adolescence and into adulthood [4]. The social consequences are evident in the negative effect of overweight on school performance and educational attainment, largely due to its detrimental effects on cognitive function among children and adolescents [5]. The economic burden is also substantial, as the health consequences of childhood overweight translate into significantly higher medical costs for healthcare systems. A systematic review and meta-analysis aimed to estimate the total medical costs associated with childhood overweight, indicated that children with overweight from high-income countries (e.g., Australia, Canada, Europe, Japan and United States) presented significantly higher per capita healthcare costs compared to those with healthy weight [6]. For children, overweight was linked to an average increase of US $237.55 in annual health expenses relative to healthy-weight peers. In addition, average hospitalization costs reached nearing US $1975 with hospital stays extended by 0.28 days [6]. Given the rising prevalence of childhood and adolescent overweight in LatAm, along with their associated health and economic impacts, there is an urgent need for comprehensive and effective public health interventions and policies to address this growing epidemic.

In LatAm, addressing childhood and adolescent overweight presents a particular challenge due to its coexistence with persistent stunting and micronutrient deficiencies [7]. This coexistence of undernutrition and overweight or obesity within individuals, households, or populations is referred to as the double burden of malnutrition (DBM) [8]. For decades, the primary focus of most LatAm countries was on combating undernutrition, often to the neglect of interventions targeting other forms of malnutrition, such as overweight [7]. The DBM complicates policy and programmatic responses, highlighting the urgent need for integrated, context-specific strategies, such as double-duty actions, which simultaneously address multiple forms of malnutrition across the spectrum. For example, interventions that reduce both childhood stunting and adult obesity, or programs that improve micronutrient deficiencies while also preventing excess calorie intake [7,9].

Research highlights that overweight is strongly influenced by the broader food environment, which shapes dietary behaviors from an early age [10,11,12]. Obesogenic environments, characterized by high availability, aggressive marketing, and affordability of energy-dense and nutrient-poor foods, play a central role in promoting excess weight gain across populations [10,11,12]. While individual factors such as physiological processes, food preferences, and physical activity patterns interact with these environments, it is increasingly recognized that structural influences on food access and choice are primary drivers of the obesity epidemic [10,11,12].

After the early years of life, which provide the first window for establishing food preferences and other habits [13], the school environment provides the second window in which health, nutrition, and physical activity habits can be shaped [11]. Given the amount of time that children spend in school, the learning and practice of healthy habits may be promoted or discouraged [11]. Previous reviews on school-based overweight prevention programs conducted in the United States and to a lesser extent in Asia, Canada, Oceania, and LatAm (mainly Argentina, Chile and Mexico), have described positive changes in health-related behaviors, nutrition literacy, and BMI [14,15,16,17]. There are very few reviews on the effectiveness and impact of overweight in school-aged children and adolescents in LatAm.

Both international reviews and those conducted in LatAm assessing school-based interventions for the prevention of overweight consistently concluded that effective interventions are multi-component and involve multiple stakeholders within the school community (e.g., teachers and parents) [17,18,19,20,21]. These comprehensive approaches typically integrate nutrition education, physical activity promotion, and food environment modifications [18,19,20,21]. However, despite these valuable insights, existing reviews often lack detailed conclusions regarding the specific activities or implementation strategies that have been most effective. This gap limits the ability to translate evidence into actionable policies and practices tailored to diverse school settings, particularly in resource-constrained regions. Moreover, previous reviews in LatAm have primarily focused on describing interventions, without examining how their design and implementation align with existing school food policies and broader regional priorities. To our knowledge, this is the first review to assess not only the effectiveness of school-based interventions for overweight prevention in LatAm, but also to evaluate the extent to which current policies in the region reflect and incorporate the best available evidence. Therefore, the aim of this article is to describe the effectiveness of school-based interventions for overweight prevention in LatAm and whether current policies, programs and other initiatives in the region align with the best available evidence.

## 2. Materials and Methods

### 2.1. Literature Search

**Figure 1:**
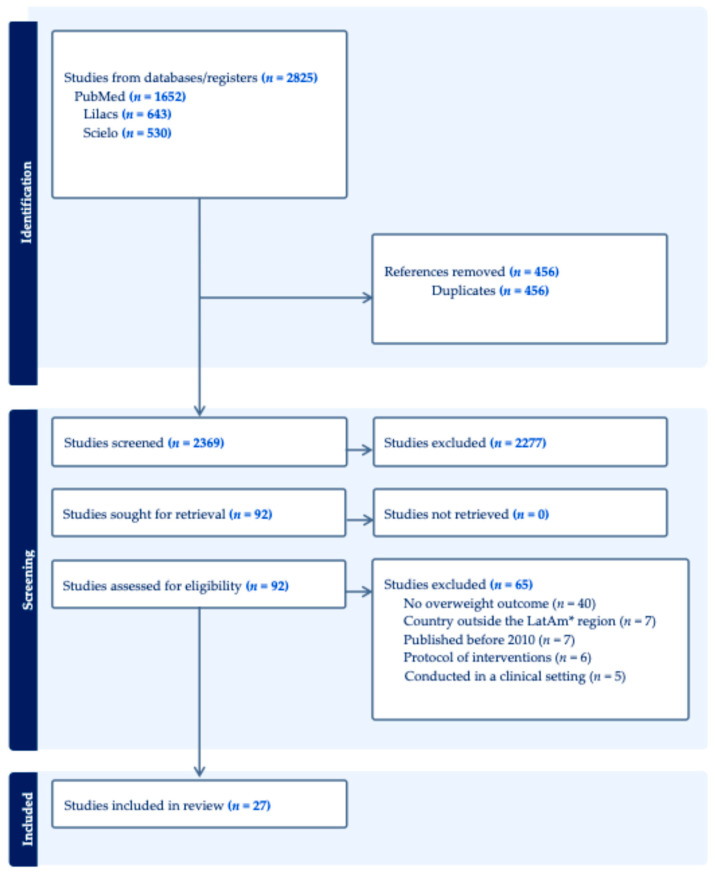
Flow diagram of 27 studies addressing school-based interventions for overweight prevention in Latin America (*LatAm) from PubMed, Lilacs and SciELO, years 2010 to 2022.

Studies published between 2010 and 2022 were identified through searches of the online databases PubMed/MEDLINE, LILACS, and SciELO. The search strategy included the following terms in English and Spanish: (school OR schoolchildren OR preschool children OR adolescents) AND (health AND nutrition OR nutritional status OR nutrition education OR diet OR physical activity OR health promotion) AND (health impact OR impact evaluation OR intervention OR law OR policy OR regulation OR norm) AND (Latin America OR South America OR Central America). Gray literature and unpublished government reports were not included in this review. This decision was made to focus exclusively on peer-reviewed publications, thereby ensuring methodological rigor, transparency, and comparability across studies. Articles published in English, Spanish, or Portuguese were eligible for inclusion. The search was conducted by six researchers who had been trained by a senior investigator with expertise in reviews. Subsequently, the two researchers demonstrating the highest concordance with the senior investigator (≥80%) were selected to independently screen the identified records by title and abstract. These researchers then assessed the full texts to determine eligibility according to predefined inclusion and exclusion criteria. During abstract screening, systematic reviews were also consulted to identify references to original studies that may not have been captured in the initial search. Finally, a third researcher reviewed the search results to ensure that no potentially relevant studies had been overlooked (Figure 1). An internal protocol was developed and approved by the research team to ensure quality and rigor.

Studies were excluded if they did not include an overweight-related outcome, were conducted in countries outside of Latin America, including the Caribbean, were published before 2010 or after 2022, described intervention protocols rather than implemented interventions, or were carried out in clinical settings. Data extraction was performed by one trained researcher using a standardized Microsoft Excel worksheet. Extracted information included: first and last authors, year of publication, country, sample size, age of child and adolescent participants, study design, intervention components, personnel delivering the intervention, intervention duration, target population, and outcomes with main findings. Interventions were classified as effective if they resulted in a statistically significant difference in BMI change between intervention and control groups. The synthesis of results is presented in tables.

### 2.2. Search Strategy: Existing Policies, Programs and Other Initiatives in LatAm

Existing policies, programs and other initiatives included school-based policies, programs, laws, decrees and government-led initiatives implemented in LatAm countries for the prevention of overweight. To identify those focused on school settings, an exhaustive search was conducted using publicly available information from 19 countries in the LatAm region. The search was originally conducted between 2017 and 2018 and was updated in July 2025. Information sources included official government materials (such as websites of ministries and agencies responsible for health and education). The search and data extraction were performed by three researchers and validated by a senior expert. All policies, programs, and initiatives were summarized in tables specifying the country, source of information, and their alignment with previously identified overweight prevention strategies.

## 3. Results

### 3.1. Literature Search

The literature search yielded 2825 publications across the selected databases (Figure 1). After removal of duplicates and screening of titles and abstracts, 92 articles remained. Full-text review and application of the eligibility criteria resulted in the exclusion of 65 articles: 40 that did not report overweight-related outcomes, seven describing school-based programs conducted outside of LatAm, seven published outside the specified time frame, six that were intervention protocols only, and five conducted in clinical settings. Ultimately, 27 studies met the inclusion criteria and were included in the review.

### 3.2. General Characteristics of the Studies

Among the 27 interventions included in the review, most were conducted in Chile (41%), employed a pre–post intervention design (41%), adopted a preventive approach (85%), and were reported as effective (52%). The most frequently incorporated components of effective school-based interventions were physical activity (93%) and nutrition literacy (86%) (Table 1).

### 3.3. Effective School-Based Interventions for Overweight Prevention

Effective interventions comprised several components, identified as follows: nutrition literacy, physical activity, nutritious foods and diets, provision of free and safe drinking water and healthy food environment [22,24,26,27,29,30,32,35,36,37,39,40,43,47].

Effective interventions to reduce BMI were characterized by the inclusion of at least three of four key components. These typically involved nutrition literacy, physical activity, nutritious foods and diets, and healthy food environment. For example, in the intervention by Alvirde-García U. (2013) [35], activities included changes to the school curriculum, development of educational materials, increased time dedicated to physical activity, family-based exercise, provision of a list of healthy foods to school kiosk vendors and expanded availability of healthy food options. After three years, reductions in BMI were observed (Table 1). Although only two studies incorporated the healthy food environment component, these interventions demonstrated the strongest reductions in BMI [27,40]. All but one of the effective BMI reduction interventions included in this analysis were multicomponent. The exception was the study by Soto-Sánchez J. (2014) [24], in which the only subcomponent implemented was active recess, corresponding to the component of physical activity.

Overall, effective BMI reduction interventions ranged from 12 to 36 months in duration, with the target population participating one to two times per week or up to five times per week, accompanied by continuous monitoring. These interventions were delivered by trained personnel, such as nutritionists or teachers instructed by nutritionists, with active participation from various members of the school community, including students, parents, teachers, and school store staff.

Although effective interventions share some common elements and activities, there is heterogeneity across studies in terms of secondary activities.

#### 3.3.1. Nutrition Literacy

The nutrition literacy component was consistently integrated into effective school-based interventions alongside other elements, most often in combination with physical activity. Table 2 shows that the component of nutrition literacy component consists of three activities: modifying the school curriculum, designing educational material, and training the academic community. However, some of these activities included various secondary activities. For example, in Mexico, Shamah (2012) [40] distributed healthy recipe calendars aimed at parents and conducted puppet theater for children. In Chile, Kain (2010) [32] developed educational materials related to healthy eating habits aimed at children. Two interventions with the same component, nutrition literacy, included designing educational materials, but the secondary activities differed.

#### 3.3.2. Physical Activity

The physical activity component was one of the most frequently incorporated element in effective school-based interventions. These interventions generally combined physical activity strategies with nutrition literacy (Table 1). Enhancements to physical activity opportunities included initiatives such as promoting active play before and after school or during recess, as well as creating supportive environments through the development of playgrounds and recreational spaces (Table 2). In addition, interventions often integrated high-quality physical activity into the school curriculum, with classes taught by educators specifically trained in physical education (Table 2).

#### 3.3.3. Nutritious Foods and Diets

The nutritious foods and diets component was incorporated into four effective interventions and was often implemented in combination with nutrition literacy and physical activity (Table 1). Specific strategies included providing school store concessionaires with a list of approved healthy foods that could be sold, offering a nutritionally balanced school breakfast, and prohibiting the sale of unhealthy foods (e.g., sugar-sweetened drinks, ultra-processed products) (Table 2). In addition, some interventions promoted the use of food labeling as a tool to help children identify and select healthier options (Table 2).

#### 3.3.4. Provision of Free and Safe Drinking Water

The component of providing free and safe drinking water (e.g., water that is free from microbial contamination and suitable for drinking) was included in only one intervention, Shamah T. (2012) [40], alongside nutrition education, physical activity, nutritious foods and diets, and a supportive healthy food environment. This strategy aimed to ensure that schoolchildren had access to free, safe drinking water and to encourage its daily consumption; however, the intervention did not specify a recommended amount of water intake.

#### 3.3.5. Healthy Food Environment

The healthy food environment component was implemented in two interventions, each in combination with other strategies and these interventions demonstrated the strongest reductions in BMI as mentioned earlier. In one study, Rinat (2013) [27], the implementation of a healthy food environment was paired with nutrition literacy and physical activity, while in the other, Shamah (2012) [40], it was combined with nutrition education, physical activity, nutritious foods and diets, and provision of free and safe drinking water (Table 1). This component focused on modifying the school food environment by increasing the availability of healthy foods and beverages, such as fruits and vegetables, within the school store and prohibiting the sale of sugary drinks, ultra-processed foods, and items high in sodium, sugar, fat, or calories (Table 2). In addition, efforts were made to ensure that healthy foods and beverages were more broadly accessible within the school setting (Table 2).

### 3.4. Existing Policies, Programs and Other Initiatives Aiming at Preventing Child Overweight in LatAm

**Figure 2:**
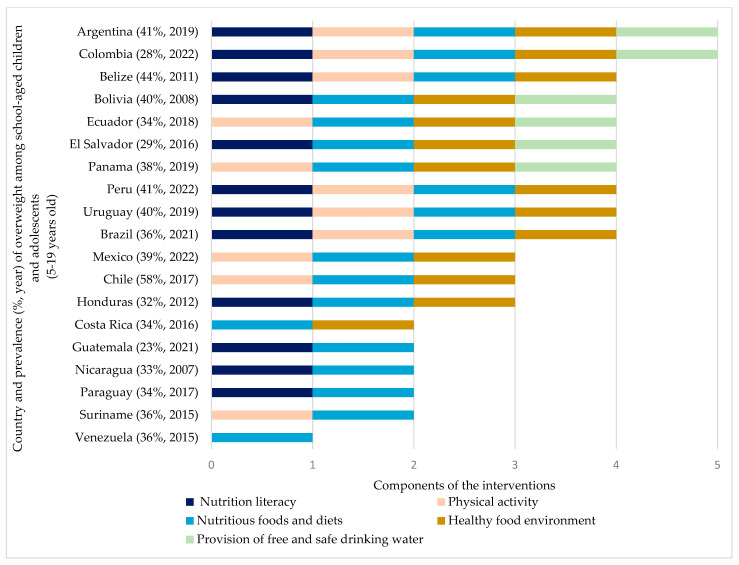
Existing school-based policies, programs and other initiatives in LatAm countries and inclusion of effective components for the prevention of overweight ^1^ in children and adolescents. ^1^ Country and prevalence of overweight among school-aged children and adolescents aged 5–19 y data from UNICEF, The State of the World’s Children 2024 Statistical Compendium [50].

Table S1 presents school-based policies and programs currently implemented in the LatAm region. Figure 2 shows that while most countries lack comprehensive, multi-component strategies to prevent overweight, 19 countries have adopted at least one of the evidence-based components. Five countries have implemented two components. Guatemala, Nicaragua, and Panama have adopted nutrition literacy alongside the nutritious foods and diets. Suriname has focused on physical activity and nutritious foods and diets. Costa Rica has implemented nutritious foods and diets and introduced the healthy food environment. Three countries have incorporated three components. Chile and Mexico have implemented the physical activity, nutritious foods and diets, and a healthy food environment. In contrast, Honduras has implemented nutrition literacy, nutritious foods and diets, and the healthy food environment. Eight countries (Belize, Bolivia, Brazil, Ecuador, El Salvador, Panama, Peru, and Uruguay) have implemented four components, among these, the nutritious foods and diets and implementation of a healthy food environment were consistently included.

Argentina and Colombia have adopted policies, programs and other initiatives that address all five components as part of their school-based strategies.

## 4. Discussion

In LatAm countries, effective school-based interventions for overweight prevention are multi-component, integrating nutrition literacy, physical activity, nutritious foods and diets, provision of free and safe drinking water and healthy food environments. These interventions operate at multiple levels, aiming to influence behaviors among students, teachers, and parents, with trained personnel playing a central role in implementation. The duration of effective interventions ranged from 6 to 36 months. While all countries examined in this review have included at least one of these components in their policies, programs, or initiatives; only Argentina, Chile, Colombia, Mexico, Peru, and Uruguay have adopted comprehensive multi-component strategies supported by a formal regulatory framework. In the LatAm region, a clear gap remains between countries that align their school programs with evidence-based practices and those that do not. A critical concern is that several programs in the region are temporary and vulnerable to discontinuation. To enhance sustainability, these programs should be embedded within regulatory frameworks that include clear accountability, monitoring, enforcement mechanisms, and sufficient funding. Policymakers are encouraged to design child overweight prevention strategies grounded in the best available scientific evidence to ensure maximum effectiveness.

Inclusion of multiple components reflect that effective school-based interventions are part of a broader systems approach, which underscores the interdependence of multiple sectors in improving school-age children nutrition, particularly the food and education systems [49]. The education system provides a powerful platform for nutrition promotion via school curricula that teach healthy eating, physical activity, and school food environments that offer safe water and nutritious options [36]. Simultaneously, the food system is responsible for ensuring that nutritious, safe, affordable, and sustainable foods are available and promoted through supply chains, fortified foods, healthy food environments, and supportive policies [49]. Together, these coordinated interventions demonstrate how aligning the efforts of the education and food systems can create enabling environments that support healthy growth and development among schoolchildren [49]. The effective components found in this review act synergistically to influence children’s behaviors, food choices, and energy balance.

Nutrition literacy helps students develop beliefs about healthy eating habits and adopt healthier dietary behaviors [51]. A systematic review conducted in 2019 found that nutrition education for children positively influences their food preferences, self–efficacy, nutrition knowledge, physical activity levels, and leads to improvements in BMI z–scores and waist circumference. Providing nutrition education to the entire school community requires significant economic resources [52]. However, economic evaluations have found school-based interventions to be cost-effective strategies for preventing chronic diseases in the population [52]. In the long term, these interventions can lead to healthier and more productive children and adults, ultimately contributing to an increase in national gross domestic product [52].

The physical activity component was one of the most frequently observed component in effective school-based interventions. School offer multiples opportunities for children to be physically active, such as during recess, sports activities, and physical education classes [53]. Evidence suggests that improvements in these school-based settings can contribute up to 50% of the daily physical activity recommended for children and adolescents [53]. Despite this potential, the prevalence of insufficient physical activity among adolescents aged 11–17 in LatAm countries remained high in 2016; approximately 80% among boys, and 89% among girls [53].

The effectiveness of access to nutritious foods and diets can be attributed to the fact that children and adolescents consume between one-third to one-half of their daily meals at school, making it a crucial setting for interventions aimed at improving dietary habits [54]. A systematic review by Micha et al. found that school dietary policies helped to increase fruit intake by 0.27 servings/d and vegetable intake by 0.04 servings/d [54]. Additionally, children reduced their consumption of sugar-sweetened beverages (SSB) by 0.18 servings/d and unhealthy snacks by 0.17 servings/d [55]. School meals standards also led to reductions in total fat intake by approximately 1.5% of daily energy, saturated fat intake by 1%, and sodium intake by 170 mg/d [50]. These policies not only enhance dietary quality during childhood but may also have lasting benefits into adulthood [50].

Evidence shows that changes in the food environment can help prevent overweight. Anderson et al. found that every 10% point increase in access to junk food at school was associated with a 1% increase in children’s BMI [55]. Also, low availability of certain foods, such as fruit and vegetable, is linked to higher odds of childhood overweight [52]. Interventions that limit students’ access to SSB, vending machines, and other unhealthy food items sold in school stores have the potential to reduce their calorie intake [56]. Studies suggest that a low energy-dense diet can lower BMI and reduce childhood overweight, and that changes to the school food environment positively influence both BMI and dietary behaviors [56].

Likewise, ensuring access to safe drinking water (e.g., water that is free from microbial contamination and suitable for drinking) can contribute to the prevention of overweight by promoting water as the preferred beverage choice. This component is effective because substituting SSB with water reduces overall total energy intake, leading to potential health benefits [57].

Interventions also addressed parental and school community participation (e.g., schoolchildren, parents, teachers, and school store personnel), corroborating findings from other international reviews [58,59,60]. Parental involvement has been recognized as having a fundamental impact on changing children’s lifestyle behaviors and preventing overweight, as noted in both international reviews [61,62] and studies conducted in LatAm [61].

Bautista-Castano et al. [63] found that interventions lasting between 6 and 12 months were more effective than shorter or long-term interventions. A systematic review conducted in LatAm reported that the duration of three effective interventions ranged from 6 to 11 months [64]. Additionally, four effective interventions identified in this review that lasted less than 6 months shared a common physical activity component and were conducted either by study staff or by teachers trained in physical activity. Studies conducted among populations in Europe and the United States have shown that physical activity interventions lasting at least 13 weeks were associated with decreases in BMI and reductions in adipose tissue [65,66].

Only six countries, namely Argentina, Chile, Colombia, Mexico, Peru, and Uruguay have implemented effective multi-component strategies within a regulatory framework (e.g., laws passed by a government) in combination with front-of-pack warning label systems (FOPWL). The FOPWL is implemented typically in the form of octagons with “High in” or “Excess in” labels. The implementation of FOPWL has emerged as a transformative policy tool for improving food environments across LatAm [67], indicating that these six countries have demonstrated a broader commitment to comprehensive, multisectoral approaches [67]. By clearly identifying products high in critical nutrients such as sugar, sodium, and saturated fats, FOPWL not only empowers consumers to make healthier choices but also incentivizes the food industry to reformulate products [67]. The convergence of school-based and population-wide regulatory interventions, such as warning labels and healthy school food policies, strengthens the public health response to poor nutrition, a key driver of non-communicable diseases in the region

Although some governments in LatAm have introduced regulatory initiatives to improve food environments, most of these initiatives lack a strong foundation in scientific evidence [68]. Countries such as Costa Rica, Guatemala, Paraguay, Suriname and Venezuela have taken steps to address nutritional deficiencies through school feeding programs that include the component of nutritious foods and diets. Even though these initiatives have not yet integrated the full set of key components identified in this review to comprehensively target overweight prevention, it is a positive step in that direction.

Despite the high prevalence of overweight across LatAm, with 31% of children aged 5–19 years affected (49 million including the Caribbean) [69], our review shows that only Argentina, Chile, Colombia, Mexico, Peru and Uruguay have implemented regulations that promote multi-component policies within a regulatory framework. For example: Argentina includes multi-component policies, programs and other initiatives in Law No. 3704 [70], which promotes a varied and healthy diet for school-aged children and adolescents, Law No. 26396 [71], which addresses the prevention and control of eating disorders, and Resolution 732/2016 [72], which established the National Program for Healthy Eating and Obesity Prevention. Chile includes multi-component strategies in its school feeding program [73] and regulates food composition and advertising through the Food Law No. 20.606 [74] and the Food Nutrition Labeling Manual [75]. Colombia incorporates multi-component strategies through policy instruments, including Resolution No. 2492 [76], which regulates the implementation of the FOPWL, guidelines for regulating food sales in schools [77], and the promotion of healthy environments under the Law No. 2120 [78].

Mexico has modified the NOM-051-SCFI/SSA1-2010 [79], which mandates FOPWL to inform consumers about the presence of critical nutrients in pre-packaged foods and beverages. Additionally, Mexico has updated the national guidelines regulating the sale and distribution of prepared and processed foods and beverages within schools of the National Educational System [80]. Peru has implemented Law No. 30021 [81], which promotes healthy eating among children and adolescents. Uruguay has enacted Law No. 19.140 [82] for Healthy Eating in Educational Centers, implemented a school feeding program [83], and issued guidelines for food sales and advertising in both public and private primary and secondary schools [82]. These policies address the prevention of overweight while also targeting nutritional deficiencies.

In LatAm, important gaps exist between countries that are aligned with effective, evidence-based school interventions and those that are not. One reason for this gap is the continued lack of recognition of the DBM, the coexistence of undernutrition and overweight, as a serious public health problem in the region [7]. Historically, programs have focused primarily on addressing nutritional deficiencies and stunting [7]. However, current policies, programs and other initiatives have not been effectively adapted to the rapid rise in overweight, while the reduction in undernutrition has slowed [7,8]. This review highlights the potential for school-based interventions to address the DBM in LatAm. Multi-component interventions, combining nutrition literacy, physical activity, nutritious foods, healthy food environments, and provision of free and safe drinking water can serve as double-duty actions. For example, nutrition literacy in Mexico and Chile [32,40] teaches balanced diets that support growth while preventing excess weight, and provision of micronutrient-rich foods in countries like Costa Rica [84] and Guatemala [85] can improve nutritional status without promoting overweight. Country-level strategies show that integrating multiple components, as seen in Argentina and Colombia, provides a framework to simultaneously reduce micronutrient deficiencies, and overweight.

Sustaining school-based interventions and policies for overweight prevention in LatAm faces several challenges, including short political cycles, limited or inconsistent financing, challenges in enforcement, particularly when programs are not protected by a regulatory framework, such as a law [86]. These factors can undermine the continuity and effectiveness of programs over time. Successful mechanisms for sustainability, however, demonstrate that these challenges can be addressed [86]. For example, Chile’s Food Law has established a regulatory framework for school food environments, supported by monitoring and enforcement systems to ensure long-term implementation of nutrition standards [87]. Its key mechanisms include FOPWL for unhealthy foods, restrictions on marketing to children, bans on the sale of unhealthy foods within schools, and government-led inspections and penalties to maintain compliance [87]. Integrating multi-component interventions into national curricula, securing stable funding, and involving stakeholders across government, schools, and communities are additional strategies that can enhance the durability and impact of school-based policies in the region [86].

It is important to note that overweight is a multifactorial condition, and its determinants extend beyond BMI-based outcomes to include dietary patterns, lifestyle behaviors, and the broader school and social environments in which children live. For example, a cross-cultural study in Latin America preschool children found that physical fitness, screen time, and diet were strongly associated with abdominal obesity (measured by waist circumference and waist-to-height ratio), beyond what BMI alone indicated [88]. Furthermore, BMI has well-recognized limitations as an indicator of adiposity, as it does not differentiate between fat mass and lean mass and may vary in its interpretation across sexes and ethnic groups due to differences in stature, body composition, and growth trajectories. For instance, a study examining racial-ethnic disparities in obesity in the United States found that, even at the same BMI levels, Mexican-American and non-Hispanic Asian children exhibited higher body fat percentages and greater risk for metabolic syndrome compared to other racial/ethnic groups. This underscores the limitations of BMI as a sole indicator of adiposity across diverse populations [89]. In this review, BMI-for-age z-scores from the WHO Growth Reference (5–19 years) were used, given their international comparability and wide acceptance for monitoring child and adolescent growth in population-based research and intervention settings. Nonetheless, caution is warranted when generalizing BMI-based findings across diverse populations.

A major strength of this study is that, to our knowledge, it is the first review that describes the specific policies, programs and other initiatives comprising effective components for the prevention of overweight within the school environment across the LatAm region, and compare the current existing policies, programs and other initiatives with existing evidence-based recommendations.

Nonetheless, this study has several limitations. A limitation of this review is the exclusion of gray literature and unpublished government reports. While this approach enhanced methodological rigor and ensured comparability across studies, it may have restricted the scope of evidence by omitting region-specific practices, policy documents, and program evaluations that are not available in peer-reviewed sources. Also, the heterogeneity in the activities within each component makes it difficult to determine which specific activity should be implemented region-wide; however, countries can select from the list of activities those which best suit their context and current resources. Additionally, it is possible that relevant policies and interventions may have been adopted after our search period ended, however, policy implementation is often a lengthy process. Although BMI-for-age z-scores from the WHO Growth Reference were used as the outcome measure, BMI has limitations as a proxy for adiposity and may vary in interpretation across sexes and ethnic groups. Future research in Latin America should seek to incorporate complementary measures such as waist circumference, skinfolds, or other body composition assessments, as well as a deeper examination of cultural and environmental factors that shape overweight risk.

## 5. Conclusions

Effective school-based interventions for preventing child overweight in LatAm are multi-component and include a combination of nutrition literacy, physical activity, nutritious foods and diets, healthy food environment, and provision of free and safe drinking water (e.g., water that is free from microbial contamination and suitable for drinking). These interventions are also multi-level, targeting behavior changes among schoolchildren, teachers, and parents, as well as modifying the school environment. Trained staff play a key role in implementing overweight prevention activities within schools.

Notably, only Argentina, Chile, Colombia, Mexico, Peru and Uruguay have integrated effective multi-component approaches within a regulatory framework. A major concern is that most countries rely on programs that operate only for limited periods and face the risk of discontinuation. It is recommended to ensure the sustainability of these interventions by embedding them within regulatory frameworks with clear accountability and monitoring and enforcement measures and ensure adequate financing. Policymakers should base child overweight prevention strategies on the best available scientific evidence to maximize effectiveness.

## Figures and Tables

**Table 1:** Characteristics of studies conducted in school settings in Latin America, organized by country (from most to fewest articles) and by year of publication (most recent first).

Author (Year) Country	*n*	Age in Years	Design	Intervention Components	Delivered by	Duration of Intervention	Target Population	Outcomes	Main Findings
Ximena Diaz Martínez et al. (2015) [22] Chile	306	5–7	Pre and post intervention evaluation [Prevention]	Nutrition literacy and physical activity	Physical education teachers, teachers trained by nutritionist and nutritionist or study staff	5 months, nutrition workshops: 1 every month and physical activity workshops: 1 time/day (45 min each session)	Schoolchildren, teachers and parents	BMI Mean of pre and post ± Standard deviation	Significant reduction in BMI in boys Pre intervention = 17.7 ± 2.5, post intervention = 17.4 ± 2.4, *p* value = 0.018
Fernando Vio et al. (2014) [23] Chile	817	3–9	Pre and post intervention evaluation [Prevention]	Nutrition literacy	Teachers trained by nutritionist, nutritionist or study staff, social worker, and chef	A school year	Schoolchildren, and teachers	BMI Median (25th–75th percentile)	No significant improvement in BMI. Intervention group = 16.5(15.3, 18.3) Control group = 16.5(15.3, 18.4) *p* value > 0.05
Johana Patricia Soto-Sánchez et al. (2014) [24] Chile	156	7–15	Controlled trial (quasi-experimental) [Prevention]	Physical activity	Nutritionist or study staff	3 months, 5 times a week	Schoolchildren	BMI Median (25th–75th percentile)	Significant reduction in the prevalence of nutritional status Overweight: Pre intervention = 20 (12.8%) Post intervention = 9 (5.8%) *p* value < 0.001 Obesity: Pre intervention = 115 (73.7%) Post intervention = 99 (63.5%) *p* value < 0.001
Juliana Kain et al. (2014) [25] Chile	1474	6–8	Randomized controlled trial (experimental) [Prevention]	Nutrition literacy and physical activity	Physical education teachers, teachers trained by nutritionist	12 months, twice a week	Schoolchildren, teachers and parents	BMI Mean ± Standard deviation	No significant improvement in BMI Boys: Intervention group = 18.3 ± 2.7, Control group = 18.7 ±3, *p* value > 0.05 Girls: Intervention group = 18.3 ± 2.9, Control group = 18.3 ±2.6, *p* value > 0.05
Fabían Vásquez et al. (2013) [26] Chile	120	8–13	Pre and post intervention evaluation [Treatment]	Nutrition literacy and physical activity	Nutritionist or study staff and psychologists	3 months, 3 times a week	Schoolchildren	BMI Z-score Mean ± Standard deviation	Significant reduction in BMI Intervention group: −0.2 ± 0.4 Control group: +0.1 ± 0.4 *p* value < 0.01
Rinat Ratner et al. (2013) [27] Chile	2527	6–10	Pre and post intervention evaluation [Prevention]	Nutrition literacy, physical activity and healthy food environment	Physical education teachers, and nutritionist or study staff	24 months, 12 sessions	Schoolchildren	Prevalence of obesity and Delta of BMI z-score	The prevalence of obesity decreased significantly. Pre intervention = 23.4% Post intervention = 20.1% *p* value < 0.01 The 75% of schoolchildren with obesity and 60.5% decrease the BMI z-score −0.1 and −0.3 standard deviation, respectively
Lobos Fernández et al. (2013) [28] Chile	796	4–10	Pre and post intervention evaluation [Prevention]	Nutrition literacy and physical activity	Teachers trained by the study staff and Physical education teachers	24 months	Schoolchildren, and teachers	BMI Mean ± Standard deviation	No significant improvement in BMI Pre intervention = 18.1 ± 2.8 Post intervention =19 3 ± 2 *p* value = 0.8
Kain et al. (2012) [29] Chile	2039	6–12	Controlled trial (quasi-experimental) Pre and post intervention evaluation [Treatment]	Nutrition literacy and physical activity	Nutritionist or study staff	24 months	Schoolchildren and teachers	BMI z-score Delta of BMI	Significant reduction in BMI Boys: Intervention group = 0.53 ± 0.95, Control group = 0.71 ± 1, *p* value > 0.05 Girls: Intervention group = 0.58 ± 0.9, Control group = 0.72 ± 0.9, *p* value < 0.001
Juliana Kain et al. (2012) [30] Chile	597	4–7	Pre and post intervention evaluation [Prevention]	Nutrition literacy, physical activity and nutritious foods and diets	Physical education teachers and teachers trained by nutritionist	36 months, education in nutrition each 15 days and physical activity 4 h a week	Schoolchildren and teachers	BMI z-score Delta of BMI	Significant reduction in BMI among schoolchildren with obesity Pre intervention = 2.73 Post intervention = 2.41 *p* value < 0.0001
Fernando Vio et al. (2011) [31] Chile	1556	5–8	Controlled trial (quasi-experimental) [Prevention]	Nutrition literacy	Teachers trained by nutritionist and nutritionist or study staff	12 months, once a month	Schoolchildren, teachers and parents	Prevalence of obesity	No significant improvement on BMI Intervention group: Baseline = 19.6%, follow-up = 19.4%, *p* > 0.05 Control group: Baseline = 21.2%, follow-up = 21.5%
Kain Juliana et al. (2010) [32] Chile	741	4–10	Controlled trial (quasi-experimental) [Prevention]	Nutrition literacy and physical activity	Teachers trained by nutritionist	24 months	Schoolchildren, and teachers	BMI Mean ± Standard deviation Prevalence of obesity	Significant reduction in BMI Pre intervention = 1.03 ± 1.1, Post intervention = 0.92 ± 1, *p* value < 0.05 Significant reduction on the prevalence of obesity among females Pre intervention = 20.2, Post intervention =18.3%, *p* value = 0.03
Ernestina Polo-Oteyza et al. (2016) [33] Mexico	1888	6–11	Pre and post intervention evaluation [Prevention]	Physical activity	Physical education teachers	8 months, 5 times a week	Schoolchildren, teachers	BMI Odds ratio	No significant improvement in BMI Boys = 0.98 (0.78–1.23), *p* value = 0.876 Girls = 1.01 (0.80–1.27), *p* value = 0.959
María del Carmen Morales-Ruán et al. (2014) [34] Mexico	1020	10–13	Randomized controlled trial (experimental) [Prevention]	Nutrition literacy and physical activity	Nutritionist or study staff	6 months, 5 times a week	Schoolchildren, teachers and parents	BMI Odds ratio	No significant improvement in BMI Effect of intervention= 1.030 (0.833–1.200), *p* value = 0.708
Alvirde-García U. et al. (2013) [35] Mexico	1224	7–11	Randomized controlled trial (experimental) [Prevention]	Nutrition literacy, physical activity and nutritious foods and diets	Teachers trained by nutritionist and nutritionist or study staff	36 months, once or two times a week	Schoolchildren, teachers and parents	BMI Delta of BMI	Significant reduction in BMI Intervention group = 1.6 kg/m^2^ Control group = 1.9 kg/m^2^, *p* value < 0.01
Víctor Ríos-Cortázar et al. Mayo (2013) [36] Mexico	538	7–12	Pre and post intervention evaluation [Prevention]	Nutrition literacy	Nutritionist or study staff	-	Schoolchildren	BMI Prevalence	Significant reduction in BMI Pre intervention = 13.3% Post intervention = 8.3% *p* value < 0.001
L. Elizondo-Montemayor et al. (2013) [37] Mexico	96	6–12	Pre and post intervention evaluation [Treatment]	Nutrition literacy, physical activity and nutritious foods and diets	Nutritionist or study staff	10 months,	Schoolchildren	BMI Delta of BMI percentiles	Significant reduction in BMI There was a 2.84 [95% confidence interval (CI) = 4.10 to 1.58; *p* < 0.01] significant decrease in body mass index percentile and in body-fat percentage (95%CI = 3.31 to 1.55; *p* < 0.01)
Elizondo-Montemayor et al. (2014) [38] Mexico	304	14–16	Controlled trial (quasi-experimental) [Prevention]	Nutrition literacy and physical activity	Nutritionist or study staff	1 school year	Schoolchildren, teachers and parents	Prevalence of overweight and obesity	No significant changes in the prevalence of overweight and obesity Obesity: Pre intervention = 9% vs. Post intervention = 11%, *p* value = 0.12. Overweight: Pre intervention = 16% vs. Post intervention = 15%, *p* value = 0.86
Bacardí-Gascon et al. (2012) [39] Mexico	532	7–9	Randomized controlled trial (experimental) [Prevention]	Nutrition literacy and physical activity	Teachers trained by nutritionist, nutritionist or study staff, and Physical education teachers	24 months	Schoolchildren, teachers and parents	BMI Delta of BMI	Significant improvement in BMI At six months BMI differences between control and intervention group were −0.82, 95%CI (−0.97, 0.67) (*p* = 0.0001)
Shamah T. et al. Gomez (2012) [40] Mexico	1019	7–13	Randomized controlled trial (experimental) [Prevention]	Nutrition literacy, physical activity, nutritious foods and diets, provision of free and safe drinking water and healthy food environment	Nutritionist or study staff	6 months	Schoolchildren, teachers, parents and store personnel of the school	BMI Probability of obesity	Significant reduction in BMI. The estimated probability of obesity between baseline and the final stage for the intervention group decreased 1% (Initial estimated probability = 11.8%, 95%CI 9.0–15.2, final = 10.8, 95%CI 8.4, 13.
Margie Balas-Nakash et al. (2010) [41] Mexico	319	8–12	Pre and post intervention evaluation [Prevention]	Physical activity	Physical education teachers	6 months, 5 times a week	Schoolchildren, Physical education teachers	BMI Delta of BMI	No significant improvement in BMI Intervention group: −0.09 (−0.96 70 -0.84) Control group: −0.67 (−0.17 to 0.05) *p* value 0.675
Diana B. Cunha et al. (2013) [42] Brazil	478	10–12	Randomized controlled trial (experimental) [Prevention]	Nutrition literacy and nutritious foods and diets	Teachers trained by nutritionist and nutritionist or study staff	9 months, once a week	Schoolchildren, teachers and parents	BMI Mixed models	No significant improvement in BMI (β = 0.003; *p* = 0.75)
Marcio Atalla et al. (2018) [43] Brazil	3592	6–17	Pre and post intervention evaluation [Prevention]	Nutrition literacy and physical activity	Teachers trained by physical activity specialists	7 months	Schoolchildren, teachers and parents	BMI Z-score Delta of BMI	Significant improvement in BMI. Participants with overweight and obesity decreased BMI z-score (−0.08; 95%CI:−0.11–0.05; *p* < 0.001)
Karine Brito et al. (2019) [44] Brazil	895	14–15	Randomized controlled trial (experimental) [Prevention]	Nutrition literacy	Nutritionist or study staff	12 months, once a week	Schoolchildren, teachers and parents	BMI Mean ± Standard deviation	No significant improvement in BMI Intervention group: Baseline Mean = 20.56 ± 4.35, follow-up= 20.95 ± 4.17, Control group: Baseline Mean = 20.22 ± 3.89, follow-up = 20.61 ± 3.79 *p* value = 0.293
Ribeiro Michele et al. (2019) [45] Brazil	2276	10–13	Randomized controlled trial (experimental) [Prevention]	Nutrition literacy	Teachers trained by nutritionist	1 school year	Schoolchildren, teachers and parents	BMI Delta of BMI	No significant improvement in BMI BMI increased more in the intervention group than in the control group (Δ = 0.3 kg/m^2^; *p* = 0.05) with a greater decrease in %body fat among boys (Δ = −0.6%; *p* = 0.03) in the control group.
Juan Pablo Aparco et al. (2017) [46] Peru	696	7–8	Controlled trial (quasi-experimental) [Prevention]	Nutrition literacy and physical activity	Physical education teachers and nutritionist or study staff	12 months	Schoolchildren, Physical education teachers	BMI Difference in difference	No significant improvement in BMI −0.04 (95%CI −0.11; 0.03) *p* > 0.05
Diana Carolina Preciado-Martínez et al.Martínez et al. (2016) [47] Colombia	1003	6–17	Controlled trial (quasi-experimental) [Treatment]	Physical activity	Physical education teachers	2 months, twice a week	Schoolchildren, Physical education teachers	BMI Z-score Mean ± Standard deviation	Significant improvement in BMI without clinic relevance Girls: Pre intervention = 0.033± 1.15, post intervention = 0.80 ± 0.22, *p* value = 0.03 Boys = Pre intervention = 0.000 ± 0.99, post intervention = −2.25 ± 0.91, *p* value = 0.01
Rausch Herscovici et al. (2013) [48] Argentina	369	9–11	Randomized controlled trial (experimental) [Prevention]	Nutrition literacy, physical activity and healthy food environment	Nutritionist or study staff	6 months	Schoolchildren and parents	BMI One-way ANOVA	No significant improvement in BMI Differences between intervention and control groups Girls = 0.02 (–0.27; 0.30) *p* value = 0.9 Boys = −0.15 (–0.37; 0.07) *p* value = 0.19

**Table 2:** Components and activities of effective school-based interventions for the prevention of overweight in LatAm countries.

Components	Definition	Activities
Nutrition literacy	Policies and programs that integrate nutrition and physical activity into school curricula to enhance children’s and adolescents’ knowledge, skills, and healthy behaviors, while building the capacity of teachers and staff to deliver them effectively [49].	- Modify the school curriculum to include healthy eating and nutrition topics, for example: sources of nutrients and types of food.
		- Design educational material for children and parents to use at school or at home. This material should include topics related to nutrition and healthy eating habits, for example: understanding nutritional labeling; fruit and vegetable intake; and drinking water ^1^. Likewise, playful activities should be used to teach this information (e.g., stories, brochures, refrigerator magnets, cookbooks, coloring sheets and theatrical plays).
		- Train the educational community ^2^: interactive workshops or talks should be conducted to increase knowledge and skills in food and nutrition topics (e.g., cooking workshops and talks about fruit and vegetable intake). Additionally, information and communications technologies could be used to share this information.
Physical activity	Involves incorporating physical education into school curricula and using communication strategies that encourage active living and regular participation in physical activity [49].	- Promote physical activity through campaigns that include social events with famous athletes. In addition, spaces to walk and play sports must be established within the school. Likewise, to encourage physical activity, posters related to this topic should be posted at school.
		- Provide physical education classes with certified teachers to develop activity plans for schoolchildren to improve their strength, agility, endurance, and speed. Additionally, physical education class must be part of the school curriculum.
		- Train the educational community: give training talks or interactive workshops related to physical activity using information and communication technologies. Additionally, perform family activities, such as walking or cycling.
		- Increase the time of physical activity as part of the school curriculum, providing one or two extra classes of physical education. In addition, promote active recess with dance and music, competitions and games that improve children’s strength, agility, endurance, and speed.
		- Availability of items for physical activity: materials or items to be used in physical education class or during school recess, for example: balls, jump ropes, cones, hula hoops and mats to perform sit-ups.
		- Provide infrastructure to do exercises: improvement or construction of infrastructure or esthetics of the school to perform physical activity, for example: construction basketball courts, soccer fields, and flat spaces to perform resistance exercises, speed, and agility.
		- Provide a physical activity plan.
Nutritious foods and diets	Refer to policies and programs aimed at improving dietary quality in middle childhood and adolescence. These include providing safe and nutritious school meals, promoting the use of fortified foods in schools [49].	- Provide a healthy food list for school store concessionaires to know what foods they can sell.
		- Provide a healthy school breakfast with optimal nutritional content for proper development of schoolchildren, for example: a breakfast that includes fruits and vegetables without sugary drinks.
		- Prohibit the sale of unhealthy foods at school, for example: sugary drinks, ultra-processed foods, and foods high in sodium, sugar, fat, and/or calories.
		- Use the nutritional labeling of foods to select healthy food.
Provision of free and safe drinking water^1^	Provision of clean and safe water within school settings to support children’s hydration and overall dietary quality [49].	- Provide access to free and safe drinking water^1^ to schoolchildren and encourage their daily use.
Healthy food environment	Refer to policies and guidelines that ensure access to nutritious, safe, affordable, and sustainable foods, as well as safe drinking water ^1^. This includes protecting children from marketing of unhealthy foods, promoting appropriate food labeling, and using regulatory measures to encourage healthy dietary choices among school-age children and adolescents [49].	- Change foods and beverages at the school store: increase the availability of healthy foods and beverages within the school store, such as fruits and vegetables. Additionally, prohibit the availability of sugary drinks, ultra-processed foods, and foods high in sodium, sugar, fat and/or calories. - Make healthy foods and beverages available within the school.

^1^ Water that is free from microbial contamination and suitable for drinking. ^2^ Educational community: schoolchildren, teachers, parents, entities and links of the Ministry of Education.

## Data Availability

The data on existing school-based actions in Latin American (LatAm) countries, as well as the inclusion of effective components for the prevention of overweight and obesity in children and adolescents, are available in the Supplementary Materials associated with this article.

## Supplementary Materials

The following supporting information can be downloaded at: https://www.mdpi.com/article/10.3390/nu17213435/s1, Table S1: Existing school-based policies, programs and other initiatives in Latin America (LatAm) countries and inclusion of effective components for the prevention of overweight in children and adolescents.

## Abbreviations

The following abbreviations are used in this manuscript:

BMI	Body Mass Index
WHO	World Health Organization
SD	Standard deviation
LatAm	Latin America
DBM	Double burden of malnutrition
SSB	Sugar-sweetened beverages
FOPWL	Front-of-pack warning label
