# Supplementary material for: School-Based Interventions to Prevent Overweight in Latin America: A Scoping Review and Policy Analysis
Source: Nutrients. 2025 Oct 31;17(21):3435. doi: 10.3390/nu17213435 (PMC12608608; doi:10.3390/nu17213435)
Supplement: Supplementary file 1 [file nutrients-17-03435-s001.zip › nutrients-3870105-supplementary.pdf]

**Table S1**. Existing school-based policies, programs and other initiatives in Latin America (LatAm) countries and inclusion of effective components for the prevention of overweight in children and adolescents

| Existing school-based policies, programs and other initiatives in LatAm | | | | | Effective components for the prevention of overweight included in the school-based policies, programs and other initiatives (x) | | | | |
|---|---|---|---|---|---|---|---|---|---|
| **Country** | **Document´s name** | **Website Link** | **Type of instrument** | **Year of initiation or modification** | **Nutrition literacy** | **Physical activity** | **Nutritious foods and diets** | **Healthy food environment** | **Provision of free and safe drinking water** |
| Argentina | Front-of-package Labeling Law | Law #27.642 | Law | 2021 | x | | x | x | |
| | Resolution 732/2016. National Program for Healthy Eating and Obesity Prevention | Resolution # 732/2016 | Law | 2016 | x | x | x | x | |
| | National Strategy for the Prevention and Control of Non-Communicable Diseases | National Strategy | Policy | 2013 | x | x | x | x | x |
| | Law No. 3704 "Promotion of a varied and safe healthy diet for children and adolescents of school age", (Law of Buenos Aires) | Law # 3704 | Law | 2010 | x | x | x | x | |
| | School Feeding Program | School Feeding Program Regulated by Law # 3704 | Program | Year of initiation not reported, but active in 2025 | | | x | x | |
| | Law 26396, Prevention and control of eating disorders (National Law) | Law # 26396 | Law | 2008 | x | x | x | x | |
| | Healthy School Environments Guide | Guide Regulated by Law # 26396 | Guide | Year of initiation not reported, but active in 2025 | x | x | x | x | x |
| Belize | Belize National Plan of Action for the prevention and control of non-communicable diseases | National Plan | Plan | 2013-2023 | | x | x | x | |
| | School Health and Nutrition Program | School Feeding Program | Policy | 2003 | x | x | x | | |
| Bolivia | Law No. 775, law to promote healthy eating | Law #775 | Law | 2016 | | | x | x | x |
| | Law No. 622, School Feeding within the framework of Food Sovereignty and Plural Economy | Law #622 | Law | 2014 | x | | x | | |

**Table S1**. (Continued) Existing school-based policies, programs and other initiatives in Latin America (LatAm) countries and inclusion of effective components for the prevention of overweight in children and adolescents

| **Existing school-based policies, programs and other initiatives in LatAm** | | | | | **Effective components for the prevention of overweight included in the school-based policies, programs and other initiatives (x)** | | | | |
|---|---|---|---|---|---|---|---|---|---|
| **Country** | **Document´s name** | **Website Link** | **Type of instrument** | **Year of initiation or modification** | **Nutrition literacy** | **Physical activity** | **Nutritious foods and diets** | **Healthy food environment** | **Provision of free and safe drinking water** |
| Brazil | Resolution 429 on nutrition labelling of pre-packaged foods | Resolution #429 | Resolution | 2020 | | | x | x | |
| | Resolution No. 163. Prohibition of all types of advertising directed at boys and girls | Resolution # 163 | Resolution | 2014 | | | | x | |
| | School Feeding Program | Program regulated by Law #11.947 | Program | 2009 | x | | x | | |
| | Resolution 24/2010 Provides for the offer, advertising, publicity, information and other related practices whose objective is the dissemination and commercial promotion of food | Resolution # 24 | Policy | 2010 | | | | x | |
| | Law Ban on the sale of junk food in schools No.6848 | Project Law#6848 | Law | 2002 | | | x | x | |
| | National Program for promoting physical activity | Law # 9.394 | Law | 1994 | | X | | | |
| Chile | Food Law No. 20.606 | Law # 20.606 | Law | 2016 | | | x | x | |
| | Law # 20.869 Food advertising | Law # 20.869 | Law | 2015 | | | | x | |
| | School feeding program | Program | Regulation | 2004 | | | x | | |
| | National School Physical Activity Plan | Plan | Plan | 2010 | | X | | | |

**Table S1**. (Continued) Existing school-based policies, programs and other initiatives in Latin America (LatAm) countries and inclusion of effective components for the prevention of overweight in children and adolescents

| **Existing school-based policies, programs and other initiatives in LatAm** | | | | | **Effective components for the prevention of overweight included in the school-based policies, programs and other initiatives (x)** | | | | |
|---|---|---|---|---|---|---|---|---|---|
| Country | Document´s name | Website Link | Type of instrument | Year of initiation or modification | **Nutrition literacy** | **Physical activity** | **Nutritious foods and diets** | **Healthy food environment** | **Provision of free and safe drinking water** |
| Colombia | Resolution 2492 front-of-package nutrition labeling | Resolution #2492 | Policy | 2022 (start date 2023) | | | x | x | |
| | School Feeding Program | Program | Program | Year of initiation not reported, but active in 2025 | x | | x | | |
| | Promotion of healthier food environments and NCD´s prevention (“Law of junk food”) No. 2120 | Law #2120 | Law | 2021 | x | x | x | x | |
| | Food sales regulation at schools | Regulation | Regulation | 2019 | | | x | x | x |
| Costa Rica | School Feeding Program | Law # 9435 | Law | Modification 2022 | | | x | | |
| | Regulation for the operation and administration of the food store service in public educational centers | Regulation | Regulation of law | 2011 (Modification: 2013) | | | x | x | |
| El Salvador | School Feeding and nutritional program | Program | Program | Year of initiation not reported, but active in 2025 | | | x | | |
| | Regulations for healthy school stores | Norm school stores | Norm | 2017 | x | | x | x | x |
| Ecuador | School Feeding Program | Law | Law | Year of initiation not reported, but active in 2025 | | | x | | |
| | Regulation of school food stores of the national education system | Regulation of school stores | Regulation | 2014 | | | x | x | x |
| | Food labeling in Ecuador: implementation, the results and pending actions. As part of the National Plan for Good Living in Ecuador | Food labeling regulation | Regulation | 2017 | | | | x | |
| | Learning in movement | Program | Program | Year of initiation not reported, but active in 2025 | | x | | | |
| | Regulation for the authorization and control of advertising and promotion of processed foods | Regulation #259 | Regulation | 2011 | | | | x | |
| Guatemala | School Feeding Law, Regulation # 16-2017 | School Feeding Law | Law | 2017 | x | | x | | |
| Honduras | School Feeding Law | School Feeding Law | Law | 2017 | x | | x | x | |
| | Regulation of the sale of food in governmental and non-governmental educational centers | Regulation of the sale of food | Regulation | 2016 | | | x | | |

**Table S1**. (Continued) Existing school-based policies, programs and other initiatives in Latin America (LatAm) countries and inclusion of effective components for the prevention of overweight in children and adolescents

| **Existing school-based policies, programs and other initiatives in LatAm** | | | | | **Effective components for the prevention of overweight included in the school-based policies, programs and other initiatives (x)** | | | | |
|---|---|---|---|---|---|---|---|---|---|
| Country | Document´s name | Website Link | Type of instrument | Year of initiation or modification | **Nutrition literacy** | **Physical activity** | **Nutritious foods and diets** | **Healthy food environment** | **Provision of free and safe drinking water** |
| Mexico | School Breakfast Program | School Breakfast Program | Program | Year of initiation not reported, but active in 2025 | | | x | | |
| | Guidelines for the sale and distribution of food and beverages prepared and processed in schools of the National Educational System | Regulation for the foods and beverages sale and distribution<br><br>Modification 2024 | Regulation | 2014<br>(Modification 2024) | | | x | x | x |
| | Modification to the official Mexican Standard NOM-051-SCFI/SSA1-2010, Specifications | Modification NOM-051-SCFI/SSA1-2010 | Norm | 2014<br>(Modification 2020) | | | x | x | |
| Nicaragua | Comprehensive School Nutrition Program | School Nutrition Program | Program | Year of initiation not reported, but active in 2025 | x | | x | | |
| Panama | School Meal Program | Program | Regulation | Last modification 2007 | | | x | | |
| | Guidelines for the Offer of Healthy Foods in Kiosks and Cafeterias of Educational Centers | Guidelines | Guidelines | 2018 | | | x | x | |
| | Law 75. Actions to promote adequate nutrition and a healthy lifestyle in educational centers | Law #75 | Law | 2017 | | | x | x | x |
| | Resolution 3623 for Healthy School Eating in all official and private educational centers | Resolution 3623 | Resolution | 2017 | | | x | x | |
| | Reform of Law 16 on the National Sports Institute | Law #50 | Law | 2007 | | x | | | |
| Paraguay | Law No. 5210 on School Feeding and Sanitary Control | Law # 5210 | Law | 2014 | | | x | | |
| | School Feeding Program | School Feeding Program | Program | 2014 | x | | x | | |
| Peru | School Feeding Program | School Feeding Program (Regulation # 008-2012) | Regulation | 2012 | | | x | | |
| | Program for physical activity at school | Program | Regulation | 2022 | | x | | | |
| | Law No. 30021. Law for the Promotion of healthy eating for boys, girls and adolescents | Law #30021 Resolution | Law | 2013 | x | x | x | x | |

**Table S1**. (Continued) Existing school-based policies, programs and other initiatives in Latin America (LatAm) countries and inclusion of effective components for the prevention of overweight in children and adolescents

| **Existing school-based policies, programs and other initiatives in LatAm** | | | | | **Effective components for the prevention of overweight included in the school-based policies, programs and other initiatives (x)** | | | | |
|---|---|---|---|---|---|---|---|---|---|
| Country | Document´s name | Website Link | Type of instrument | Year of initiation or modification | **Nutrition literacy** | **Physical activity** | **Nutritious foods and diets** | **Healthy food environment** | **Provision of free and safe drinking water** |
| Suriname | National Action Plan for the Prevention and Control of Noncommunicable Diseases 2015 - 2020 | Plan | Plan | 2015-2020 | | x | x | | |
| Uruguay | Regulation 272/018 Food labeling | Regulation #272/018 | Regulation | 2018 | | | x | | |
| | Law No.19.140 Healthy Eating in Schools | Law #19.140 | Law | 2013 | x | x | x | x | |
| | School Food program | School Food Program | Program | Year of initiation not reported, but active in 2025 | x | | x | | |
| Venezuela | School Food Program (Regulation 1.376, 1996) | School Food Program | Law | 2015 | | | x | | |
| | Resolution #137 labeling of foods that contain sugar, saturated fats and trans fats | Resolution #137 | Resolution | 2020 | | | x | | |
